# Effects of multimodal low-opioid anesthesia protocol during on-pump coronary artery bypass grafting: a prospective cohort study

**DOI:** 10.1186/s13019-023-02395-y

**Published:** 2023-10-06

**Authors:** Maruniak Stepan, Loskutov Oleh, Druzhyna Oleksandr, Swol Justyna

**Affiliations:** 1 Department of Extracorporeal Methods of Treatment, Heart Institute Ministry of Health of Ukraine, Bratyslavska str. 5A, Kyiv, 02166 Ukraine; 2 Department of Anaesthesiology and Intensive Care, Shupyk National Healthcare University of Ukraine, Bratyslavska str. 3 A, Kyiv, PL 02166 Ukraine; 3 Department of Respiratory Medicine, Paracelsus Medical University, Prof.-Ernst-Nathan-Str. 1, 90419 Nuremberg, Germany

**Keywords:** Coronary artery bypass grafting, Cardiopulmonary bypass, Multimodal low-opioid anesthesia protocol, IL-6, Low cardiac output syndrome, Postoperative atrial fibrillation

## Abstract

**Background:**

The most favorable anesthesia protocol during on-pump coronary artery bypass grafting (CABG) in patients with coronary heart disease remains unclear, despite previous publications regarding the interaction between anesthesia protocol and postoperative complications. The aim of the study was to compare the effect of a multimodal low-opioid anesthesia protocol (MLOP) on early postoperative complications during on-pump CABG.

**Methods:**

A single-center prospective cohort study including 120 patients undergoing on-pump CABG aged 18 to 65 years, divided into two groups according to undergoing MLOP or routine-opioid anesthesia protocol (ROP). The analyzed parameters were plasma IL-6 levels, complications, duration of mechanical ventilation, length of intensive care unit stay, and hospitalization.

**Results:**

In the MLOP group, the levels of IL-6 at the end of the surgery were 25.6% significantly lower compared to the ROP group (33.4 ± 9.4 vs. 44.9 ± 15.9, p < 0.0001), the duration of mechanical ventilation was significantly shorter (2.0 (2.0; 3.0) h vs. 4.0 (3.0; 5.0) h, p < 0.001), the incidence of low cardiac output syndrome was almost two and half times lower (7 (11.7%) vs. 16 (26.7%), p = 0.037), and also the incidence of postoperative atrial fibrillation was significantly lower (9 (15.0%) vs. 19 (31.7%), p = 0.031).

**Conclusion:**

Our study confirms that using MLOP was characterized by significantly lower levels of IL-6 at the end of surgery and a lower incidence of low cardiac output syndrome and postoperative atrial fibrillation than ROP.

**Trial registration:**

The study is registered in clinicaltrials.gov №NCT05514652.

## Introduction

Ischemic heart disease is a common heart condition and a major cause of death worldwide [1]. Currently, drugs, bypass surgery, and endovascular interventions, including balloon dilatation and stenting, are used to treat coronary heart disease. The most effective are endovascular and surgical treatment, and patients who received on-pump coronary artery bypass grafting (CABG) have significantly lower 5-year mortality compared with a single use of drug therapy [2].

Despite the improvements in the monitoring of vital functions of the body and decreasing hospital mortality after CABG (up to 3.2%) [3], complications during and after cardiac surgery in patients with coronary heart disease remain present. Safaie et al. reported a postoperative complication rate of more than 20% during the hospital stay [4]. These complications relate to surgical techniques, anesthesia, and cardiopulmonary bypass protocols [5]. Further, contact of blood components with the surface of the extracorporeal circuits, reperfusion injuries, endotoxemia, and surgical trauma are among the processes that can activate the pro-inflammatory system and cytokine release, mainly interleukin-6 (IL-6) during on-pump CABG [6, 7]. IL-6 is a widely discussed biomarker in conjunction with extracorporeal circulation and inflammation during cardiac surgical interventions [8]. Recent investigations confirmed the interaction between the immune system and the opioids, both in terms of stimulatory and suppressive effects, but this is still not clearly understood [9].

This study aimed to compare the effects of multimodal low-opioid protocol (MLOP) with routine-opioid anesthesia protocol (ROP) on early postoperative complications during on-pump CABG.

## Methods

This pilot prospective single-center cohort study was conducted at the Heart Institute Ministry of Health of Ukraine, Kyiv, Ukraine, between 01.01.2019 and 24.02.2022. The recruitment of patients was carried out from February 2019 to August 2020.

**Fig. 1:**
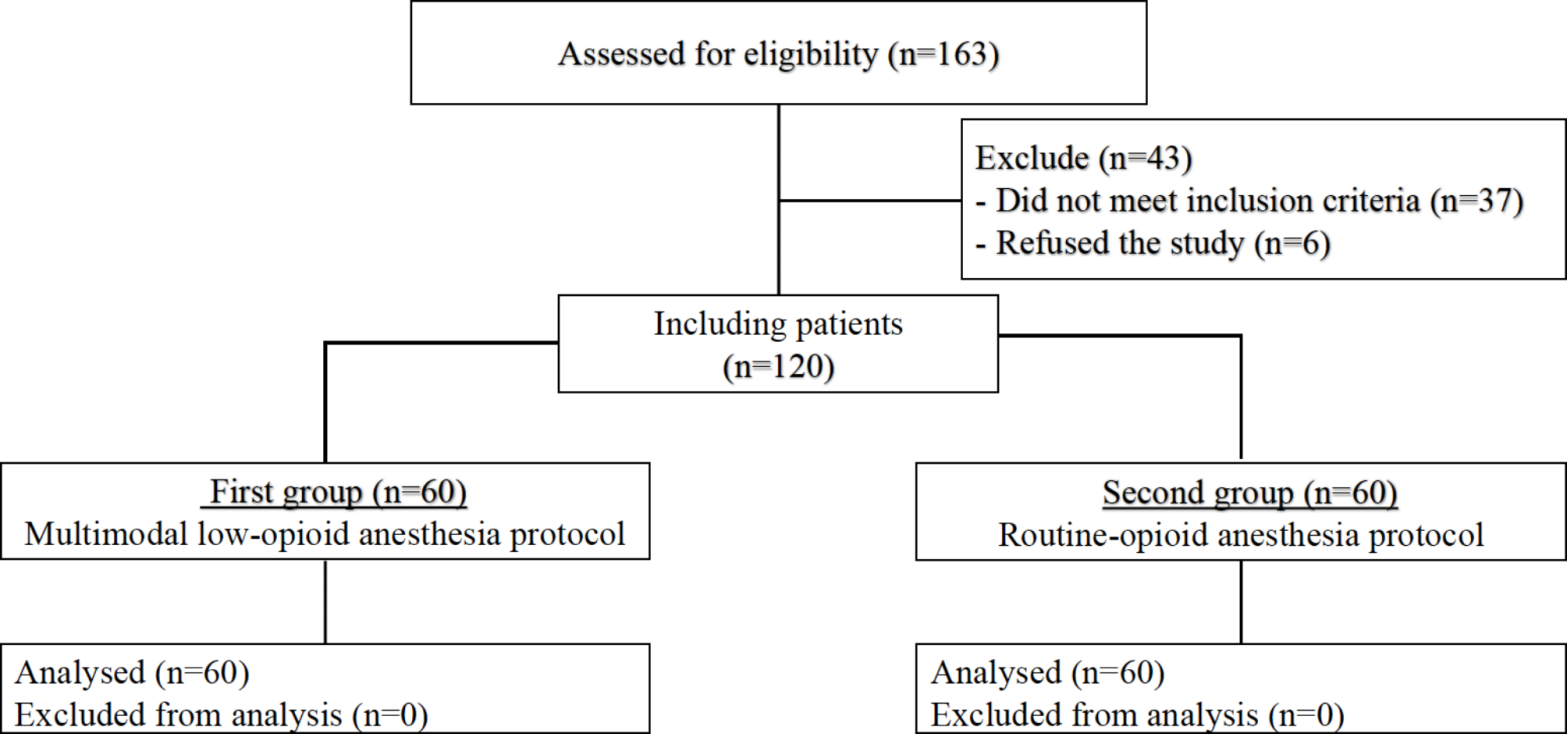
Flow chart diagram of inclusion criteria and number of patients

Patients aged 18 to 65 years, with an ejection fraction > 30% and a perioperative risk assessment for EuroSCORE II < 5%, who underwent on-pump CABG, were included. One-hundred-twenty patients who met the inclusion criteria and agreed to participate in the study were divided into the MLOP group (n = 60) or the routine opioid protocol (ROP) group (n = 60) (Fig. 1). Exclusion criteria were patient refusal, off-pump CABG, and the need for additional intraoperative intervention on the heart, which proved to be.

The study was performed in compliance with the “Rules of ethical principles of scientific medical research with human participation”, approved by the Declaration of Helsinki. Each patient signed informed consent to participate in the study. The study was approved by the Ethics Committee of P.L. Shupyk National Health Care University of Ukraine (Protocol №10 from November 5, 2018). The study is registered in clinicaltrials.gov №NCT05514652. The manuscript adheres to the STrengthening the Reporting of OBservational studies in Epidemiology (STROBE) Statement (Supplemental file).

### Anesthesia and surgery

Intraoperative monitoring included electrocardiogram, invasive blood pressure, central venous pressure, oxygen saturation, bispectral index (BIS), end-tidal partial pressure of carbon dioxide, end-tidal concentration of sevoflurane, nasopharyngeal temperature, and urine output.

MLOP provided for induction of anesthesia with intravenous (iv) propofol administration using the dosage of 1.5–2 mg/kg at 40 mg in intervals of 10–15 s, iv fentanyl dosage 1–1.5 µg/kg and iv pipecuronium bromide dosage of 0.1 mg/kg. Before intubation, a lidocaine 1 mg/kg bolus was added intravenously, with the simultaneous establishment of a continuous infusion at a dose of 1.5–2 mg/kg/h. Lidocaine infusion was continued during the surgery until the patient was admitted to the intensive care unit. After intubation and before the surgical incision, all patients were administered a bolus of ketamine (0.5 mg/kg) and were started continuous infusion of dexmedetomidine at a dose of 0.7 µg/kg/h. If indicated (increased heart rate and/or blood pressure), fentanyl was used as an additional analgesic during surgery by bolus injection (up to 1–3 µg/kg for the entire surgery).

ROP was provided for induction of anesthesia with the iv administration of propofol dosage of 1.5–2 mg/kg at 40 mg in intervals of 15–20 s, iv fentanyl at a dose of 1–1.5 µg/kg, and iv pipecuronium bromide dosage of 0.1 mg/kg. For analgesia, bolus injections of fentanyl were used at a dose of 8–10 µg/kg for the entire duration of the operation, and muscle relaxation – pipecuronium bromide at a dose of 0.1 mg/kg.

In both groups, the depth of anesthesia was monitored using the bispectral index (BIS-Vista monitor, Aspect Medical Systems, Newton, MA); the dosage of sevoflurane was titrated (from 1.5vol% to 2.5vol%) to maintain BIS values from 40 to 60. Sevoflurane was administered in the oxygenator circuit during CPB through a calibrated vaporizer.

Intraoperative mechanical ventilation (Dräger Medical Deutschland GmbH, Lübeck, Germany) targeted normoventilation under FiO_2_ 0.5, maintaining the pCO_2_ at the level of 35–40 mm Hg in arterial blood gas analysis.

In all patients, CABG was conducted using cardiopulmonary bypass (CPB). CPB was performed on the heart-lung machine “System 1” (Terumo, USA) using disposable membrane oxygenators “Inspire 6” and “Inspire 8F” (Sorin group, Italy) in mild hypothermia (+ 32 °C). A Heparin dosage of 300 IU/kg body weight was administered intravenously before CPB to achieve an activated coagulation time (ACT) greater than 480 s. ACT was measured every 30 min during CPB. After discontinuation of CPB, protamine sulfate was used to counteract the anticoagulant effect of heparin. Cannulation of the right atrium and ascending aorta were performed. The initial priming volume consisted of 500 ml of 4% Gelaspan solution (B. Braun Medical SA. Switzerland.), 100 ml of 4.2% sodium bicarbonate solution, 300 ml of 0.9% sodium chloride solution, and 100 ml of 15% mannitol solution. Red blood cell mass (RBCM) during CPB was added if the hemoglobin level was below 7.0 g/dl [10]. Oxygen delivery (DO_2_) during CPB was maintained at > 272 ml O_2_/min/m^2^.

Intraoperative myocardial protection strategy electrically included fibrillatory arrest with mild hypothermia (32 °C). Cardioplegia was not performed during CPB. Electrically induced ventricular fibrillation was performed by Fibrillator Fi 20 M, Stockert GmbH. A low-voltage generator created fibrillation (current frequency—50 Hz, current-voltage—12 volts, current strength—25 mA). The duration of a one-time clamping of the aorta did not exceed 15 min, after which at least 5 min passed before the next clamping.

### Data collection

Data collection was performed before and during surgery and in the postoperative period until the day of discharge. At the preoperative stage, demographic characteristics (age, sex, body weight), NYHA class, echocardiographic parameters (left ventricle ejection fraction (LV EF) and end-diastolic volume (EDV)), coronary angiography data, laboratory parameters (hemoglobin), and the previous history of myocardial infarction or percutaneous coronary interventions were recorded.

Intraoperative data included hemodynamic parameters, such as heart rate and blood pressure (recorded by a Philips IntelliVue MP50 patient monitor) and blood gas test (performed by ABL800 FLEX blood gas analyzer), duration of anesthesia and surgery, duration of aortic cross-clamping (XCL), and types and volumes of blood products transfused.

Blood samples were collected before the beginning of anesthesia in the preoperative room and immediately after sternum closure in the operative room. Further, blood samples were centrifuged, then plasmas were either frozen and stored at -40 °C or immediately used for IL-6, which were determined by enzyme-linked immunosorbent assay using the standard commercial IL-6 ELISA kit (Diaclone, France) according to the manufacturer’s instructions.

Data collected during the postoperative period included the duration of mechanical lung ventilation, frequency of cardiac complications (postoperative arrhythmias, low cardiac output syndrome), length of ICU stay, and hospitalization.

The decision about extubation was made regarding the following clinical criteria: full recovery from muscle relaxation and anesthesia; patients are easy to rouse, neurologically intact, lift head and sticks out tongue; stable hemodynamics (heart rate and blood pressure are within ordered parameters, bleeding is controlled); spontaneous muscle movement with stable respiratory rate and adequate oxygenation, which was confirmed by arterial blood gases (SaO_2_ > 92%, on FiO_2_ < 50%).

Postoperative complications were defined as general or specific to CABG surgery and classed according to their onset time: immediate, early, and late. The occurrence of adverse events was monitored from the beginning of anesthesia until discharge. Postoperative low cardiac output syndrome (LCOS) was defined as hemodynamic instability requiring continued after-surgery pharmacologic support with at least two inotropic agents (norepinephrine, milrinone, dobutamine) on postoperative day 1. Postoperative atrial fibrillation (POAF) was defined as clinically significant atrial fibrillation (ECG recordings on one or more ECG leads), which demonstrated the presence of atrial fibrillation characteristics ECG features lasting at least 30 s in the (intra- and) postoperative setting, which requires treatment with rate or rhythm control agents or requires anticoagulation, and/or extends the duration of hospitalization [10]. Ventricular tachycardia, ventricular fibrillation, and atrioventricular block were also registered from the beginning of anesthesia until discharge. Patients were on continuous ECG monitoring for 2 days after surgeries.

### Missing data and bias

The only missing parameters were found in blood gas analyses during surgery because there was no specific protocol for scheduled intraoperative blood sampling. The mean value substitution method was used for the imputation of the missing values. The average value was calculated over all the values available from the other waves of data collection for the same individual. Non-consecutive patients were included in the study, which depended on the availability of investigators in the hospital. Thus, selection bias of the studied subjects could be observed. This bias was eliminated by sufficient sample size for the internal validity of our study.

### Sample size

The sample size was calculated based on the frequency of occurrence of LCOS, which was taken at 5% [11]. According to the frequency of this complication, the minimal sample size in the pilot study (with a confidence interval of 95% and a margin of error of ± 5%) must include at least 59 participants [12].

### Statistical analysis

Mainly, the results were reported as mean (M) ± standard deviation (SD). In case of abnormal distribution of results, data were reported as median (Me) and 1-st (Q_25_) and 3-rd (Q_75_) quartiles—Me (Q_25_; Q_75_). In the normal distribution of data to determine the reliability of statistical indicators, the student’s t-test, and at the same time, in the absence of normal distribution, the non-parametric Mann-Whitney U-test. Pearson’s xi-square test or Fisher’s exact test (as appropriate) were used to analyze the categorical variables, such as the rate of postoperative complications between both groups. Univariate analysis, using the unpaired t-test to compare measurement data and Fisher’s exact test to compare enumeration data, was performed to assess statistically significant risk factors for LCOS, and those with P < 0.05 were then entered into a logistic regression analysis to identify the independent risk factors for LCOS (LCOS or not as an independent variable, variables with P < 0.05 obtained through univariate analysis as dependent variables). Differences at p < 0.05 (95.5%) were considered significant. The statistical data processing program “XLSTAT” was used to analyze the obtained data.

## Results

The preoperative clinical characteristics in MLOP and ROP groups were without significant differences (Table 1).

**Table 1:** Patient’s and perioperative characteristics in MLOP (multimodal low-opioid anesthesia protocol) and ROP (routine-opioid anesthesia protocol) group

Parameters		MLOP group (n = 60)	ROP group (n = 60)	p-value
Age, years		60 (56; 63)	59 (54; 62)	0.419
Sex, n (%) - men - women		44 (73.33%) 16 (26.67%)	42 (70.00%) 18 (30.00%)	0.408
Body weight, kg		95.7 ± 16.1	98.6 ± 17.3	0.345
EuroSCORE II, %		3.00 ± 1.20	3.20 ± 1.3	0.390
NYHA FC > 2 > 3		27 (45.00%) 33 (55.00%)	24 (40.00%) 36 (60.00%)	0.428
LV EF, %		46.52 ± 8.06	47.18 ± 9.61	0.681
EDV, ml		147.48 ± 20.14	145.15 ± 21.17	0.537
MI, n (%)		13 (21.67%)	11 (18.33%)	0.741
PCI, n (%)		9 (15.00%)	8 (13.33%)	0.886
AH, n (%)		40 (66.67%)	42 (70.00%)	0.723
RCA dominance, n (%)		49 (81.7%)	54 (90.0%)	0.190
CA lesion n(%)	LCMA	16 (26.7%)	22 (26.7%)	0.239
	LCA	43 (71.7%)	36 (60.0%)	0.177
	LAD	47 (78.3%)	41 (68.3%)	0.215
	RCA	38 (63.3%)	46 (76.7%)	0.111
Initial Hb, g/dL		11.9 ± 1.31	12.4 ± 1.25	0.064
Duration of operation, min		192.83 ± 14.90	194.80 ± 14.16	0.460
Duration of anesthesia, min		221.95 ± 16.61	224.88 ± 18.30	0.360
Number of anastomoses: − 2, n (%) − 3, n (%)		26 (43.33%) 34 (56.67%)	22 (36.67%) 38 (63.33%)	0.245
Duration of CPB, min		83.6 ± 12.2	83.7 ± 11.23	0.115
Duration of XCL, min		23.5 ± 5.8	25.2 ± 5.1	0.182
Needs or RBCM - 1 unit > 1 unit		22 (36.67%) 7 (11.67%)	18 (30.0%) 10 (16.67%)	0.512

Notes. FC—functional class; LV EF—left ventricular ejection fraction; EDV—end-diastolic volume; MI—myocardial infarction; PCI—percutaneous coronary interventions; AF—atrial fibrillation; AH—arterial hypertension, LMCA—left main coronary artery, LCA—left circumflex artery, LAD—left anterior descending, RCA—right coronary artery, XCL—cross-clamping; RBCM—red blood cell mass, CPB—cardiopulmonary bypass

The predicted risk of in-hospital mortality after major cardiac surgery, according to EuroSCORE, did not differ between study groups (p = 0.390). Also, perioperative characteristics, such as total duration of surgery and anesthesia, duration of CPB, and aortic XCL during the imposition of the distal end of the aorto-coronary anastomosis were similar between the groups (all p > 0.05) (Table 1). We obtained similar changes in the dynamics of the acid-base status and blood gas during CPB in each study group (Table 2).

**Table 2:** Dynamics of the parameters of the acid-base state and blood gas during CABG

Parameters	Initial values		CPB beginning		CPB end	
	MLOP	ROP	MLOP	ROP	MLOP	ROP
Hemoglobin, g/dL	11.7 ± 12.22	12.3 ± 1.34	9.72 ± 1.02*	10.26 ± 1.12*	10.5 ± 1.14	11.1 ± 1.24
Lactate, mmol/l	1.19 ± 0.31	1.18 ± 0.35	1.25 ± 0.46	1.21 ± 0.40	1.35 ± 0.38	1.34 ± 0.50
Glucose, mmol/l	5.60 ± 1.07	5.76 ± 1.14	5.29 ± 1.1	5.35 ± 1.11*	5.85 ± 1.1	5.46 ± 1.13
pH	7.36 ± 0.05	7.36 ± 0.04	7.35 ± 0.03	7.35 ± 0.04	7.36 ± 0.04	7.35 ± 0.04
p_v_CO_2_, mm Hg	36.50 (32.25; 44.75)	42.50 (37.00; 45.75)	40.00 (35.5; 42.75)	38.50 (33.00; 44.00)	40.50 (32.50; 44.00)	38.50 (32.50; 42.75)
p_v_O_2_, mm Hg	64.00 (54.25; 72.00)	63.50 (49.50; 70.00)	65.50 (46.50; 67.75)	62.50 (43.25; 73.00)	65.00 (47.25; 68.75)	63.00 (48.25; 68.75)
ctO_2_, Vol %	15.91 ± 1.64	16.76 ± 1.79	13.31 ± 14.38*	14.00 ± 1.49 *	14.38 ± 1.52	15.20 ± 1.66
p50, mm Hg	29.00 (26.25; 30.75)	29.00 (26.00; 30.00)	28.00 (26.00; 30.00)	28.50 (26.00; 31.00)	29.00 (26.00; 31.00)	28.00 (27.00; 30.00)
cBase (Efc), mmol/L	-2.70 (-4.57; 2.25)	-2.60 (-3.98; 1.75)	-2.50 (-4.07; -0.73)	-2.95 (-4.27; 0.95)	-3.20 (-4.43; 1.67)	-3.10 (-4.25; 0.07)
c HSO _3_^−^ (P, st), mmol/l	22.23 ± 2.11	22.21 ± 1.65	22.03 ± 1.34	21.88 ± 1.49	21.99 ± 1.90	21.80 ± 1.37

Notes. * - p < 0.05—in comparison with initial values; pCO_2_—partial pressure of carbon dioxide in the blood; pO_2_—partial pressure of oxygen in the blood; ctO_2_—oxygen capacity of blood; p50—partial pressure of oxygen at half saturation of hemoglobin with oxygen; cBase—lack of basics; cHCO_3_^−^– concentration of hydrogen carbonate ions

**Fig. 2:**
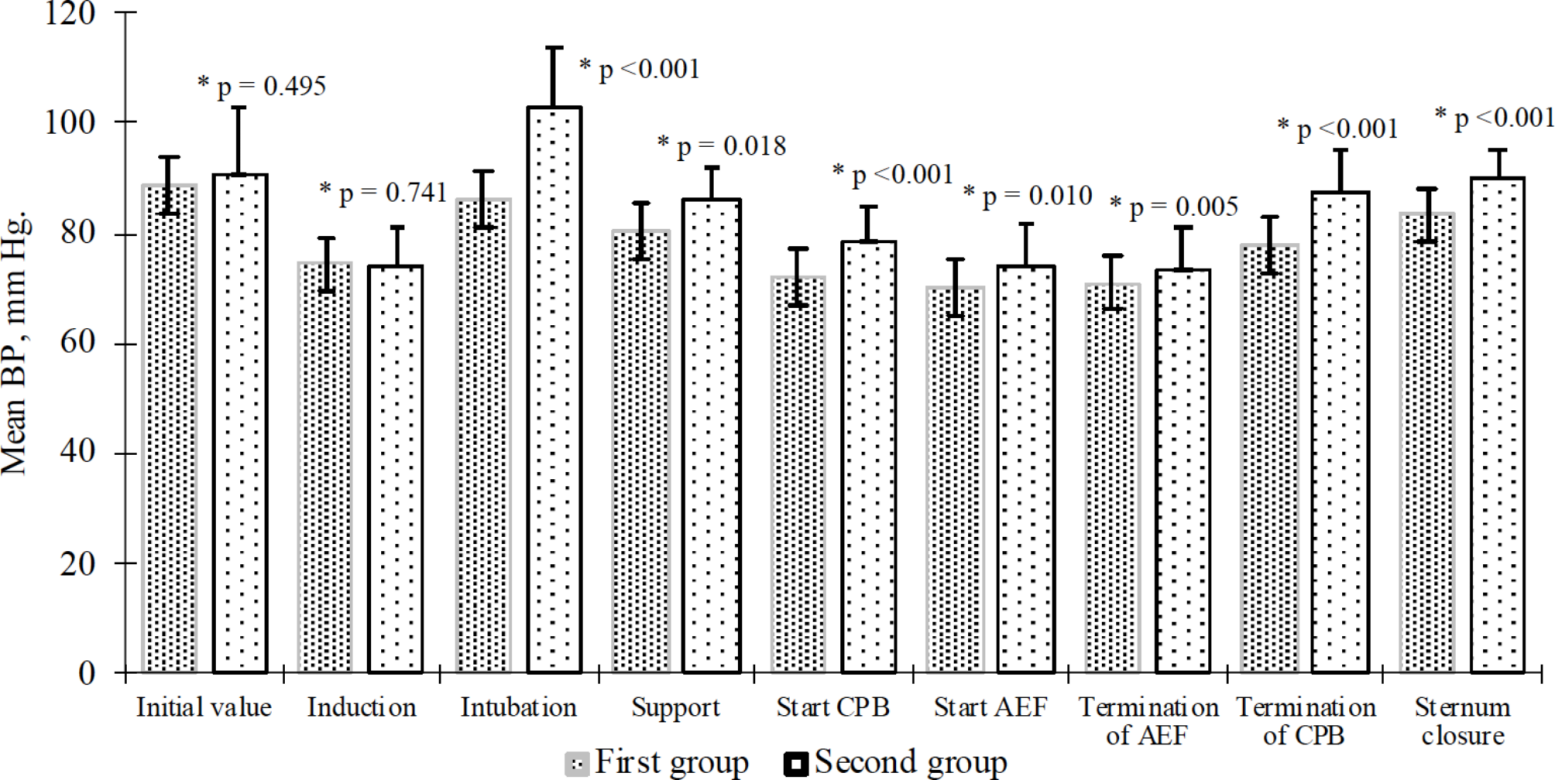
The average mean blood pressure values in patients of both groups at different stages of surgery (m ± SD). *—compared with the ROP group, BP—blood pressure, CPB—cardiopulmonary bypass, AEF—artificial electric fibrillation

The intraoperative changes in mean arterial pressure did not exceed 20% compared to baseline in both groups. But the mean blood pressure values were significantly lower in patients of the MLOP group compared to the ROP group at the time of intubation (p < 0.001), maintenance of anesthesia (p = 0.018), the beginning (p < 0.001) and end of CPB (p < 0.001), the beginning (p = 0.010) and end (p = 0.005) of electrical fibrillation and sternum closure (p < 0.001) (Fig. 2).

**Fig. 3:**
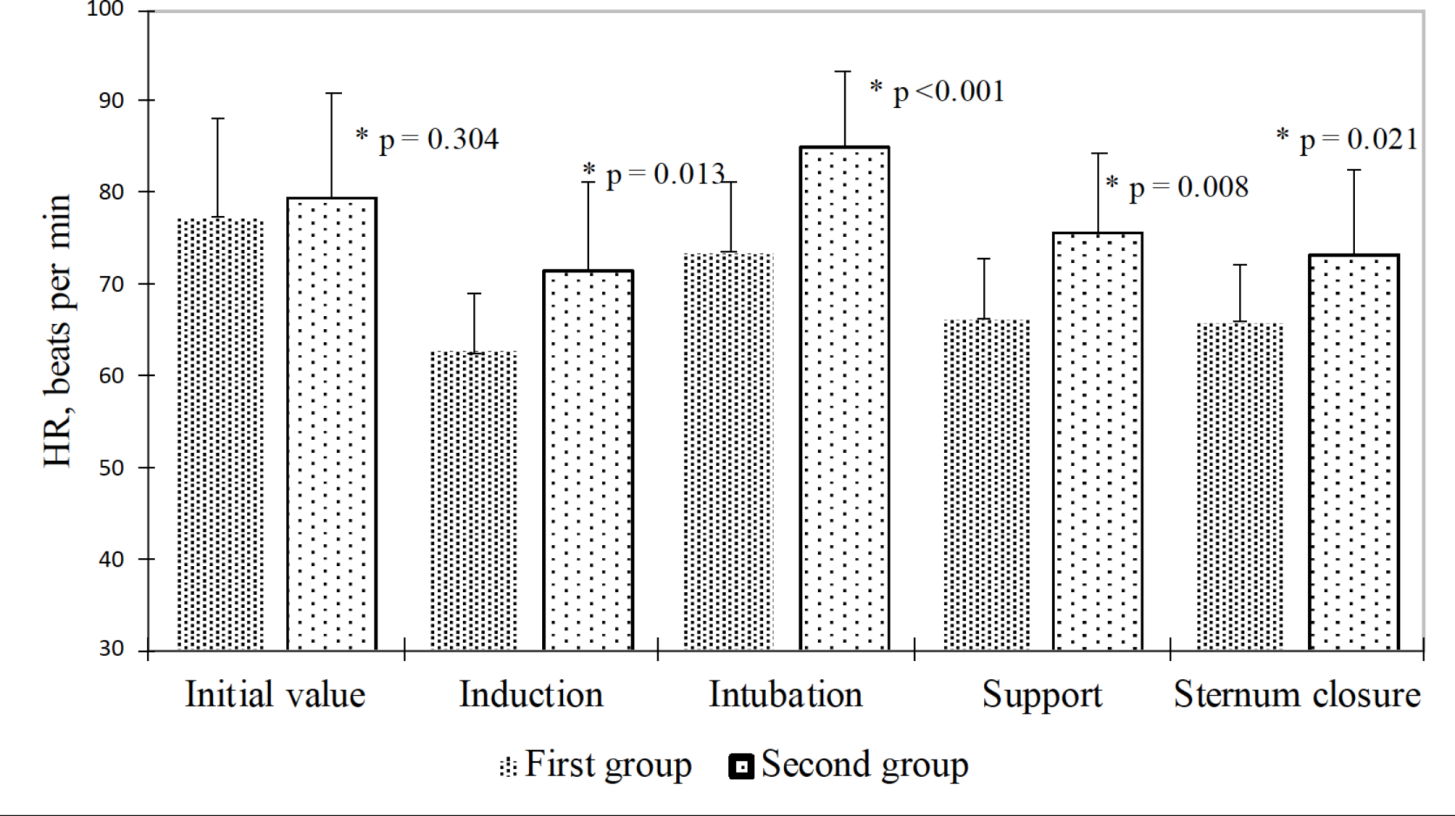
Mean heart rate values in patients of both groups at different stages of surgery (m ± SD). *—in comparison with the routine opioid protocol (ROP) group

Compared to the ROP group, the heart rate in patients of the MLOP group was significantly lower at the stages of induction (p = 0.013), intubation (p < 0.001), maintenance of anesthesia (p = 0.008), and sternum closure (p = 0.021) (Fig. 3).

**Fig. 4:**
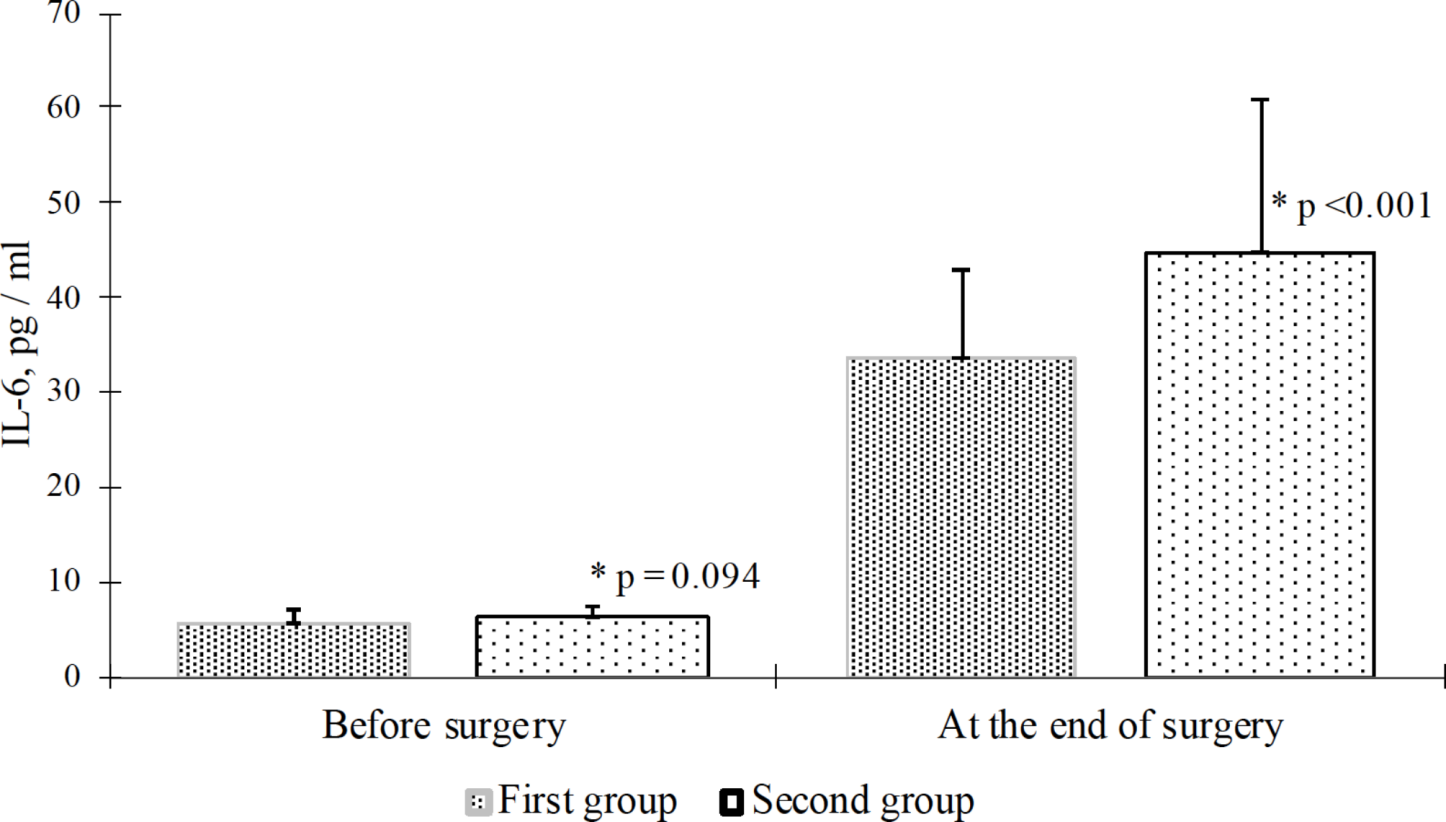
Dynamics of changes in IL-6 levels during on pump coronary artery bypass grafting depend on the anesthesia protocol (m ± SD). * - compared with the (routine opioid protocol) in the ROP group

When comparing the values of IL-6 after sternum closure between the study groups, it was found that in patients of the MLOP group IL-6 levels were significantly lower (by 25.6%) compared with the ROP group (33.4 ± 9.4 pg/ml vs. 44.9 ± 15.9 pg/ml, p < 0.0001) (Fig. 4).

In 8–12 h after surgery, troponin I levels significantly increased by 26.1% (p = 0.015) and 45.7% (p = 0.0002) in both MLOP and ROP group compare to initial values, respectively, and appeared significantly lower (p = 0.049) in MLOP compared to ROP group.

The duration of mechanical ventilation in the early postoperative period was significantly shorter in the MLOP group compared to the ROP group (2.0 (2.0; 3.0) h versus 4.0 (3.0; 5.0)) h, p < 0.001). Postoperative complications were significantly different in the POAF (p = 0.031) and LCOS (p = 0.037) between groups, but no significant differences were detected in the rate of reintubation, inotropic support > 24 h, ventricular fibrillation or tachycardia, and AV block (all p > 0.05) (Table 3).

**Table 3:** Postoperative complications in the early postoperative period

Complications		Research groups		p-value
		MLOP group (N = 60)	ROP group (N = 60)	
Reintubation	Yes	4 (6.7%)	7 (11.7%)	0.088**
	No	56 (93.3%)	53 (88.3%)	
POAF	Yes	9 (15.0%)	19 (31.7%)	0.031*
	No	51 (85.0%)	41 (68.3%)	
LCOS	Yes	7 (11.7%)	16 (26.7%)	0.037**
	No	53 (88.3%)	44 (73.3%)	
Inotropic support > 24 h	Yes	10 (16.7%)	19 (31.7%)	0.055*
	No	50 (83.3%)	41 (68.3%)	
VT or VF	Yes	2 (3.3%)	4 (6.6%)	0.402**
	No	58 (96.7%)	56 (93.4%)	
AV block	Yes	6 (10.0%)	4 (6.7%)	0.509**
	No	54 (90.0%)	56 (93.3%)	

Notes. *—Pearson’s xi-square test; **—Fisher’s exact test; LCOS—low cardiac output syndrome; POAF—postoperative atrial fibrillation; VF—ventricular fibrillation; VT—ventricular tachycardia

Mainly, the length of ICU stay of patients of the MLOP group was significantly lower compared to the ROP group (2.0 (2.0; 3.0) days versus 3.5 (3.0; 4.0) days, p < 0.001). However, the total length of stay in the hospital did not differ significantly between the study groups (11.0 (9.25; 12.75) days versus 12.0 (11.0; 13.0) days, p = 0.056). Patients with LCOS were characterized by lower LVEF before surgery, higher use of a routine opioid protocol of anesthesia, longer duration of CPB and aortic XCL, and higher levels of IL-6 at the end of surgery (all p < 0.05) (Table 4).

**Table 4:** Analysis of risk factors for developing postoperative low cardiac output syndrome (LCOS) in the early postoperative period after on pump coronary artery bypass grafting (CABG)

Factors	LCOS (N = 23)	Nо LCOS (N = 97)	p-value
Age, years (Me(Q_25_;Q_75_)	60 (58;63)	59 (55;62)	0.122
Male, n (%)	20 (86.96%)	66 (68.04%)	0.070*
EuroSCORE II, % (m ± SD)	3.41 ± 1.13	3.03 ± 1.26	0.185
AH, n (%)	15 (65.22%)	67 (69.07%)	0.721*
MI, n (%)	7 (30.43%)	17 (17.53%)	0.165**
Initial Hb, g/dl (m ± SD)	11.73 ± 1.37	12,34 ± 1,76	0.064
LV EF, % (m ± SD)	42.87 ± 8.59	47.56 ± 8.71	**0.022**
Routine-opioid protocol of anesthesia, n (%)	16 (69.56%)	44 (45,36%)	0.037**
Duration of surgery, min (m ± SD)	197.95 ± 14.96	192.83 ± 14.30	0.128
Duration of CPB, min (m ± SD)	90.39 ± 11.00	81.87 ± 11.76	**0.002**
Duration of XCL, min (m ± SD)	27.43 ± 5.07	23.74 ± 5.27	**0.003**
Needs of RBCM ≥ 1 unit, n (%)	17 (73.91%)	50 (51.55%)	0.052**
IL-6 at the end of surgery, pg/ml	53.74 ± 13.88	35.72 ± 12.03	**0.001**

Notes. *—Pearson’s xi-square test; **—Fisher’s exact test; FC—functional class; LV EF—left ventricular ejection fraction; EDV—end-diastolic volume; MI—myocardial infarction; AH—arterial hypertension, XCL—cross-clamping; RBCM—red blood cell mass; Hb—hemoglobin; CPB—cardiopulmonary bypass

Logistic regression analyses identified the following two independent predictors of LCOS: duration of CPB and level of IL-6 at the end of surgery. Table 5 presents the detailed results of the multivariable analysis.

**Table 5:** Multivariate regression results of postoperative low cardiac output syndrome (LCOS) predictors

Indicators	OR	95 CI	p
Duration of CPB, min	1.07	1.01–1.13	0.011
IL-6, pg/ml	1.10	1.05–1.16	< 0.001

Notes. CPB—cardiopulmonary bypass; IL-6—interleukin-6

## Discussion

We performed a pilot single-center cohort study of 120 patients undergoing on-pump CABG. Two anesthesia protocols, multimodal low-opioid (fentanyl 1–3 mcg/kg) vs. routine-opioid anesthesia protocol (fentanyl 8–10 mcg/kg), were compared. Both groups received inhalation anesthesia (Sevoflurane). The study patients received a bolus of ketamine and lidocaine at the beginning of the surgery with continuous infusion of lidocaine and dexmedetomidine and a lower bolus of fentanyl (to cumulative dose, 1–3 mcg/kg). The cumulative dose of fentanyl differed between twice and 4 times the low dose for the control patients. The results showed that the use of multimodal low-opioid protocol during CABG was characterized by lower levels of IL-6 at the end of surgery and was associated with a shorter duration of mechanical ventilation, lower incidence of POAF and LCOS, and shorter ICU stay.

Opioid analgesics (8–15 µg/kg throughout surgery) have been generally used in cardiac anesthesia for decades to maintain hemodynamic stability and reduce hormonal and metabolic responses to surgical stress [13]. Nevertheless, the use of opioids is also characterized by many side effects, such as the cardiodepressive effect and prolonged duration of postoperative mechanical ventilation [14]. According to Crystal at el., opioid administration may cause pulmonary vasoconstriction, which leads to decreased right ventricular function in patients after CABG [15].

Due to the negative effects of opioids, Kwanten et al. recommend a multimodal approach in analgesia in cardiac surgery patients to effectively control pain during the surgery and improve outcomes [16]. Brown et al. suggested that multimodal general anesthesia should be based on a combination of antinociceptive agents, allowing each to act on different targets in the nociceptive system [17]. Using more drugs in decreased doses enhances the desired effects while minimizing side effects [18]. According to this, important components of multimodal anesthesia are drugs that affect the central nervous system, such as dexmedetomidine and ketamine, and drugs with less specific targets, such as lidocaine [19].

Low dose opioid anesthesia has been studied previously. However, previously released meta-analysis include different key components of the anesthesia [20]. Also, our protocol is based on subnarcotic dose of ketamine, lidocaine and dexmedetomidine with additional administration of low dose of opioids. The analysis was focussed on length of stay in the ICU and in the hospital. The main limitation of this meta-analysis was missing information about cardiac complications and missing details of low opioid anaesthesia protocol, which are provided in our study.

As for the results of our study, the use of MLOP decreased the incidence of LCOS and POAF compared to the ROP, which had higher levels of IL-6 at the end of surgery.

Regarding low cardiac output syndrome (LCOS), no standardized definition of this condition exists in current literature, thus different study findings cannot be compared directly. Schoonen A, et al. in their publication summarized different criteria used for the definition of LCOS described in literature and subsequently estimated the incidence of LCOS immediately after surgery by applying these definitions to a large patient cohort [21]. They found 171 different definitions and using the 10 most frequently reported ones resulted in an estimated incidence of intraoperative LCOS.

According to the studies found in the literature, the incidence of LCOS varies between 3% and 45%, and it is associated with increased morbidity, prolongation of stay in the ICU, and increased consumption of resources [22]. In our study, the frequency of LCOS depended on the anesthesia protocol. In the MLOP group, the LCOS frequency was significantly lower than in the ROP group (11.7% vs. 26.7%, p = 0.037). The duration of CPB and the level of IL-6 at the end of surgery were two independent predictors of LCOS.

Different studies reported that postoperative atrial fibrillation (POAF) incidence ranges between 10% and 65% in cardiac surgical patients [23, 24]. This is associated with an increased rate of postoperative complications, such as congestive heart failure, renal insufficiency, thromboembolic events, and stroke, which prolong the length of hospital stay and increase rates of rehospitalization and the overall cost of hospitalization [23]. According to our study, the POAF was significantly less common in the MLOP group than in the ROP group (9 (15.0%) vs. 19 (31.7%), p = 0.031). The possible anti-arrhythmic properties of lidocaine, which blocks sodium channels in the conduction system and increases the depolarization threshold [25] and the anti-arrhythmic effect of dexmedetomidine, which could theoretically affect the transmission of sympathetic activity from the central to the peripheral nervous system [26], may also explain these results.

We also analyzed conduction disturbance between groups. There was a trend of an increased occurrence of AV block in the multimodal low-opioid protocol of the anesthesia group, which can be associated with the interaction with dexmedetomidine.

All episodes of ventricular fibrillation or tachycardia in the MLOP group and three episodes of ventricular fibrillation or tachycardia in the ROP group were recorded in the operating room after CPB termination and were successfully treated by defibrillation. These episodes could be associated with passing air through the coronary arteries. One episode of ventricular fibrillation was detected in the intensive care unit in the ROP group immediately after surgery. Coronary angiography after successful ventricular defibrillation did not reveal blood flow disturbances in the applied shunts in this case.

Our study used pro-inflammatory IL-6 to analyze the degree of inflammatory response in response to surgical stress [27]. IL-6 response is related to tissue damage, especially since it is considered an important biomarker of cardiac activity and myocardial damage, and can be related to different ways of inhibiting this response [28]. Yang et al. reported that an increase in IL-6 levels leads to a decrease in myocardial contractility, which LCOS may manifest clinically in the postoperative period [29]. Moreover, Kaireviciute et al. and Ucar et al. found that patients with POAF, who underwent on-pump CABG, were characterized by significantly higher levels of IL-6 compared with patients without the development of this complication [30, 31]. According to Bauer et al., elevated levels of IL-6 were also a predictor of prolonged mechanical ventilation and longer lengths of ICU stay [32].

Previous reports suggest that supplementing inhalatative anesthesia with conventional doses of opioids 3 or 15 mcg/kg of fentanyl did not modify the cytokine response to pelvic surgery [33].

Given the importance of inflammatory response in the development of early cardiac complications, in our study, the possible benefit in a multimodal low-opioid protocol of anesthesia is to decrease the IL-6 level, which could be achieved by the direct effect of agents such as lidocaine, ketamine, or dexmedetomidine compared to the routine protocol of anesthesia.

Previous human and animal studies suggest that all components of our multimodal low opioid protocol exert immunomodulatory and anti-inflammatory effects [9]. The results of the studies conducted by Beilin et al. demonstrated that low doses of ketamine (0.15 mg/kg) administered before the induction of anesthesia reduced the secretion of the pro-inflammatory cytokines IL-6 and TNF-alpha [34]. Similar results were shown by Roytblat L et al., which reported that a single dose of ketamine inhibited the increase in IL-6 and TNFα at 4 h after surgery [35]. Another component of our multimodal low-opioid protocol, lidocaine, has anti-inflammatory effects by blocking the EGF receptor, inhibiting IL-1 release, and improving the cytotoxic activity of neutrophils [36]. Dexmedetomidine inhibits the maturation and activity of dendritic cells by reducing the expression of I-A (b) and CD86 signaling molecules on their surface. It also limits the proliferation of helper lymphocytes and cytotoxic activity [37, 38].

Thus, in our opinion, the anti-inflammatory effect of each of the elements of our multimodal low-opioid protocol, together with better control for responses to surgical stress, could be the reason for a lower level of IL-6 at the end of the surgery in the MLOP group.

### Limitations

This study was conducted as a pilot study at a single institution, and the results are not generalizable. The low numbers in each group decreased the power of testing the primary outcome (incidence of postoperative complications) between the two groups. On-pump CABG was performed by different surgeons and cardiac surgery teams, which might also affect the study results.

The progress in surgical techniques offers new advantages replacing historical techniques. This was also confirmed by the authors of a largest retrospective observational study utilizing propensity matching of 8,875 consecutive patients, who compared in-hospital and survival outcomes between myocardial management during on-pump CABG surgery including aortic cross-clamping followed by fibrillation (XCF) and aortic cross-clamping followed by diastolic cardioplegia. They concluded, that XCF does not adversely affect in-hospital outcomes, but the long-term results demonstrate cardioplegic arrest may convey a survival advantage that would preclude routine XCF in the modern era [39]. However, XCF still offers some advantages in circumstances, when the modern techniques are not avaiable, or not affordable. The main reason for our institution performing fibrillatory arrest is to decrease the duration of surgery and the costs. Also, according to the previously mentioned observational study, XCF does not have any disadvantages such as stroke, renal failure, infection, gastrointestinal complication for in-hospital outcomes and 30-day mortality.

Our study design was based on two previous publications due to Drennan SE, et al. [40] and the second one Liu, Y. et al. [41] Clinical observations and ex vivo studies have established a strong association between inflammation and postoperative atrial fibrillation (POAF) and cardiac complications that could be related to inflammatory response. Indeed, IL-6 causes inflammation and oxidative stress, which mayresult in cardiac and cerebral injury, and has been described as a novel target for cardio- and cerebrovascular dieseases [42]. There might be an additional value of CRP, IL-10, TNF alpha for this analysis. Unfortunately, only IL-6 analysis is avaiable in the lab of our institution. However, the whole pro-inflammatory profile of cytokines was not analyzed.

Intraoperative use of TEE or any kind of hemodynamic monitoring that will show the cardiac output or cardiac index is needed during CABG. However, TEE is not established for all patients during CABG at our institution. Further, we defined LCOS as continuous hemodynamic instability requiring postoperative pharmacologic support with at least two inotropic agents (norepinephrine, milrinone, dobutamine) on postoperative day 1.

We did not analyze either the need for opioid analgesics in the early postoperative period nor assess the visual pain scale. Differences between groups regarding the incidence of postoperative delirium in relation to, for instance, the use of dexmedetomidine were not recorded.

## Conclusion

The study showed that using a MLOP during on-pump CABG was characterized by significantly lower levels of IL-6 at the end of the surgery, shorter duration of mechanical ventilation, lower incidence of LCOS and POAF, and shorter ICU stay compared to ROP.

## Data Availability

The datasets used and/or analysed during the current study are available from the corresponding author on reasonable request.

## List of abbreviations

AF: atrial fibrillation
AH: arterial hypertension
CABG: coronary artery bypass grafting
cBase: lack of basics
cHCO_3_: concentration of hydrogen carbonate ions
CPB: cardiopulmonary bypass
ctO_2_: oxygen capacity of blood
ECG: electrocardiogram
EDV: end–diastolic volume
FC: functional class
ICU: intensive care unit
LAD: left anterior descending
LCA: left circumflex artery
LCOS: low cardiac output syndrome
LMCA: left main coronary artery
LV EF: left ventricular ejection fraction
MI: myocardial infarction
MLOP: multimodal low–opioid protocol
p50: partial pressure of oxygen at half saturation of hemoglobin with oxygen
PCI: percutaneous coronary interventions
pCO_2_: partial pressure of carbon dioxide in the blood
pO_2_: partial pressure of oxygen in the blood
POAF: postoperative atrial fibrillation
RBCM: red blood cell mass
RCA: right coronary artery
ROP: routine–opioid anesthesia
VF: ventricular fibrillation
VT: ventricular tachycardia
XCL: cross–clamping
